# The Hypolipidemic Activity of Heterocyclic Thiosemicarbazones, Thioureas and Their Metal Complexes in Sprague Dawley Male Rats

**DOI:** 10.1155/MBD.1999.143

**Published:** 1999

**Authors:** Iris H. Hall, S. Y. Chen, Betsy Jo Barnes, Douglas X. West

**Affiliations:** 1 Division of Medicinal Chemistry and Natural Products School of Pharmacy University of North Carolina Chapel Hill North Carolina 27599-7360 USA; 2 Department of Chemistry Illinois State University Noemal Illinois 61761 USA

## Abstract

Heterocyclic thiosemicarbazones, thioureas and their copper, nickel, and cobalt complexes were shown to be potent hypolipidemic agents in male Sprague Dawley rats at 8 mg/kg/day, orally. These agents lowered the activity of rat hepatic rate limiting enzymes for the synthesis of cholesterol and triglycerides. The effects of these agnets on cytoplasmic ATP-dependent citrate lyase, acetyl CoA synthetase and HMG-CoA reductase activities were reduced by a magnitude to explain the reduction of serum cholesterol levels afforded by the compounds. The reduction of acetyl CoA carboxylase, *sn*-glycerol-3-phosphate synthetase and phosphotidylate phosphohydrolase activities caused by the derivatives is of sufficient magnitude to explain the observed reduction in serum triglycerides after administration of the agents.


### THE HYPOLIPIDEMIC ACTIVITY OF HETEROCYCLIC THIOSEMICARBAZONES, THIOUREAS AND THEIR METAL COMPLEXES IN SPRAGUE DAWLEY MALE RATS

Iris H. Hall[1], S.Y. Chen[1], Betsy Jo Barnes[1] and Douglas X. West[2]

[1] Division of Medicinal Chemistry and Natural Products, School of Pharmacy, University of North Carolina, Chapel Hill, North Carolina 27599-7360, USA
[2] Department of Chemistry, Illinois State University, Noemal, Illinois 61761, USA

**Abstract**
Heterocyclic thiosemicarbazones, thioureas and their copper, nickel, and cobalt complexes were shown to be potent hypolipidemic agents in male Sprague Dawley rats at 8 mg/kg/day, orally. These agents lowered the activity of rat hepatic rate limiting enzymes for the synthesis of cholesterol and triglycerides. The effects of these agnets on cytoplasmic ATP-dependent citrate lyase, acetyl CoA synthetase and HMG-CoA reductase activities were reduced by a magnitude to explain the reduction of serum cholesterol levels afforded by the compounds. The reduction of acetyl CoA carboxylase, *sn*-glycerol-3-phosphate synthetase and phosphotidylate phosphohydrolase activities caused by the derivatives is of sufficient magnitude to explain the observed reduction in serum triglycerides after administration of the agents.

### Introduction

Metal complexes containing copper, iron, cobalt, chromium, calcium and sodium of amine-carboxyboranes have previously been shown to have potent hypolipidemic activity in rodents at the low doses of 2.5 to 8 mg/kg lowering both serum cholesterol and serum triglyceride levels [1-3]. These agents modulated rat lipoproteins in a manner so that LDL-cholesterol levels were reduced whereas HDL-cholesterol levels were elevated [4], which should lead to an enhanced clearance of cholesterol from the body. These complexes suppressed LDL-binding to tissue high affinity receptor thus reducing cholesterol uptake into peripheral tissue. Yet the complexes accelerated HDL-affinity binding to hepatic receptor so that serum cholesterol could be taken up and processed to bile salts and excreted via the biliary system. These metal complexes of amine carboxyboranes also demonstrated antineoplastic/cytotoxicity and antiinflammatory activity [5,6]. The metal complexes of thiosemicarbazones have also been shown to have antineoplastic and antiinflammatory activity [7-9]. The present study involved an investigation of their potential as hypolipidemic agents in rodent.

### Methods

**Source of Compounds**
All of the derivatives [Fig 1] have been synthesized and reported in the literature previously [7].

**Hypolipidemic *in vivo* screen.**
Sprague Dawley male rats [~400g] were administered drugs prepared in 1% carboxymethylcellulose [CMC] by homogenization at 8 mg/kg/day orally for 16 days. On days 9 and 16 the rats were bled from the tail vein collecxted into capillary tubes which were centrifuged at 3000 x g for 3 min. Total cholesterol was determined on the serum by the Libermann-Burchard method [10] determined at 620 nm. Serum triglycerides were determined on day 16 using a commercial kit from Sigma Chemical, Co. which was read at 580 nm. Clofibrate at 150 mg/kg/day, orally, gemfibrozil at 90 mg/kg/day, orally and lovastatin at 8 mg/kg/day, orally were determined at their standard therapeutic dose.

**Rat Hepatic Enzyme Studies in vitro**
Enzyme assays were performed on Sprague Dawley male rats [~450g] livers homogenized [10%] in 0.025 M sucrose + 0.001 M EDTA, pH 7.2. Literature methods [11] were used to examine the effects of the compounds at 25, 50 and 100 μM over 60 min. ATP dependent citrate lyase [12], acetyl CoA synthetase [13], HMG-CoA reductase [14,15], acyl CoA cholesterol acyl transferase [16], cholesterol-7α–hydroxylase [17], acetyl CoA carboxylase [18], *sn*-glycerol-3-phosphate acyl transferase [19], phosphatidylate phosphohydrolase[20] and hepatic lipoprotein lipase [21].

### Statistic Analysis

Data displayed in the tables are the means ± standard deviations of the mean expressed as percentage of the control. N is the number of samples or animals per group. The Student's "t"-test was used to determine the probable level of significance (p) between test samples and control samples.

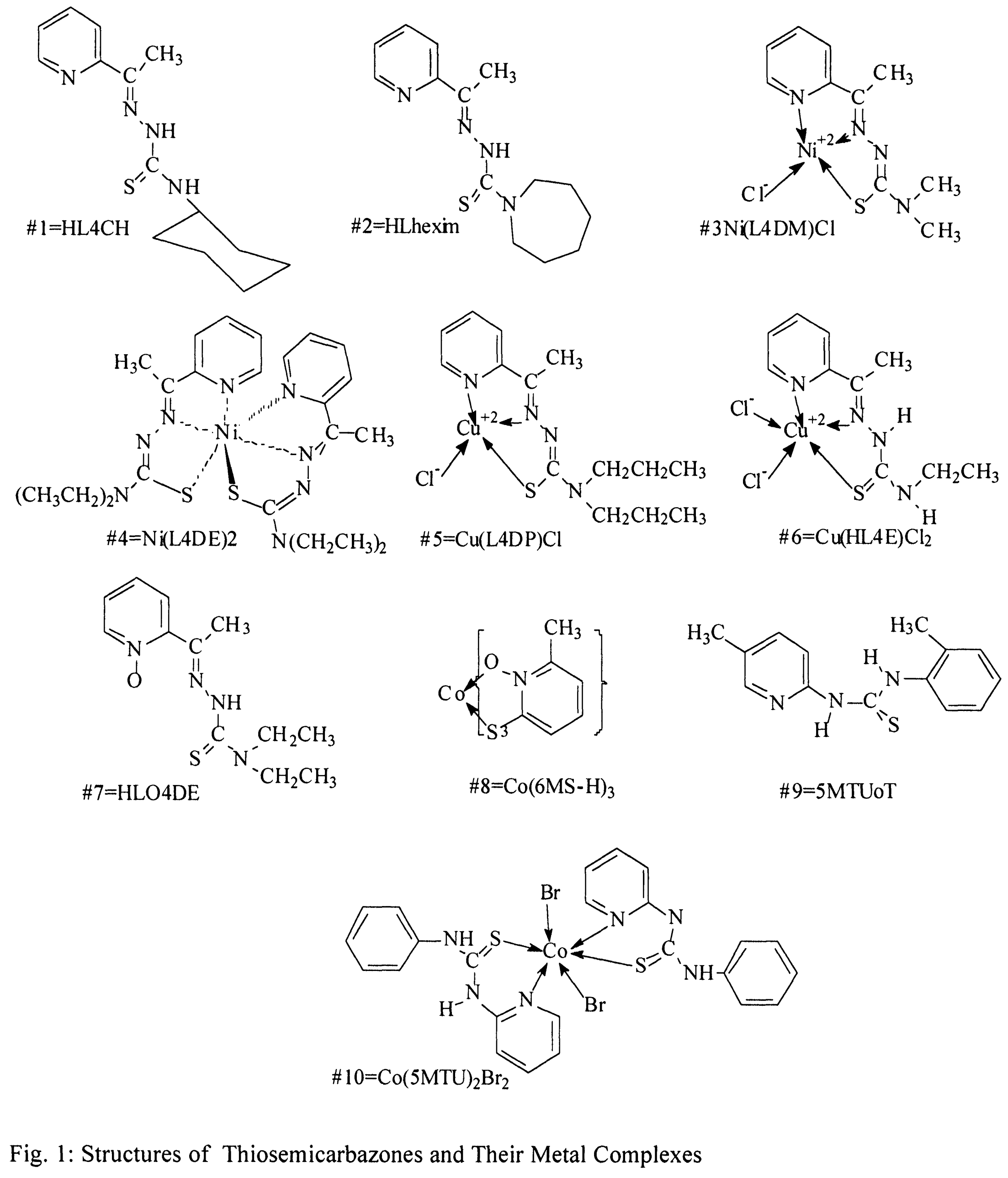

Fig. 1: Structures of Thiosemicarbazones and Their Metal Complexes

### Results

All of the thiosemicarbazones demonstrated significant hypolipidemic activity in male Sprague Dawley rats at 8 mg/kg/day orally reducing both serum cholesterol and serum triglycerides by day 16 [Table 1]. These effects were superior to the standards clofibrate at 150 mg/kg/day, gemfibrozil at 90 mg/kg/day and lovastatin at 8 mg/kg/day. On day 16 compounds **2**, **6**, **7** and **8** caused greater than 40% reduction of serum cholesterol levels while compounds **3**, **4**, **5**, **9** and **10** afforded greater than 32% reduction. Compounds **1** and **4** reduced serum triglycerides greater than 40% after 16 days and compounds **2**, **3**, **6**, **8** and **10** caused at least 35% reduction of serum triglycerides.

Table 1 Hypolipidemic Activity of Compounds **1-10** on Sprague Dawley Male Rats at 8 mg/kg/day Orally

| Compound | Serum Cholesterol | | Serum Triglycerides | |
|---|---|---|---|---|
| (N=6) | Day 9 | Day 16 | Day 9 | Day 16 |
| 1 | 81±5 | 71±3* | 76±3* | 56±4* |
| 2 | 62±4* | 59±4* | 78±4* | 64±4* |
| 3 | 93±5 | 64±5* | 72±2* | 63±5* |
| 4 | 78±6* | 61±5* | 69±3* | 59±3* |
| 5 | 71±5* | 66±6* | 82±5 | 75±3* |
| 6 | 60±4* | 55±4* | 81±3 | 61±5 |
| 7 | 52±4* | 51±2* | 84±5 | 72±5* |
| 8 | 60±3* | 57±5* | 73±4* | 65±4* |
| 9 | 82±4 | 67±3* | 79±2* | 75±4* |
| 10 | 82±5 | 62±4* | 82±4 | 62±3* |
| Clofibrate @ 150 mg/kg | 88±7 | 86±2 | 83±6 | 74±7* |
| Gemfibrozil @ 90 mg/kg | 91±5 | 82±7 | 78±6* | 62±5* |
| Lovastatin @ 8 mg/kg | 85±4 | 78±5* | 91±5 | 86±7 |
| Control -1% CMC | 100±5[a] | 100±6[b] | 100±7[c] | 100±6[d] |

a = 73 mg/dl; b = 75 mg/dl; c = 111 mg/dl; d = 112 mg/dl

The activities of the regulatory enzymes involved *in vitro* hepatic *de novo* cholesterol and triglyceride syntheses and metabolism were significantly affected by the thiosemicarbazones and their metal complexes after 60 min incubation [Table 2-4]. Acetyl CoA synthetase activity was suppressed in a concentration manner causing greater than 50% reduction at 100 μM. The inhibition of ATP-dependent citrate lyase activity followed a similar pattern of inhibition by the agents but the magnitude of reduction was a little less than with acetyl CoA synthetase activity. The activity of the regulatory enzyme of cholesterol synthesis HMG-CoA reductase was reduced in a concentration dependent manner so that greater than 50% inhibition was achieved by compounds **3, 4, 6, 7, 8, 9** and **10.** Compound **1** had the least effects on HMG-CoA reductase activity after 1 hour and also had the least ability to lower serum cholesterol levels *in vivo.* The effects of the thiosemicarbazones on cholesterol-7α-hydroxylase activity were erratic, i.e compounds **4, 9** and **10** had no effect or less than 20% reduction, compounds **1, 2, 3, 6** and **8** caused moderate reductions in activity of 24% to 41% and compound **7** afforded 62% reduction at 100 μM. Acyl-CoA cholesterol acyl transferase activity was reduced only moderately by compounds **1, 7** and **8** by approximately 32%.
Acetyl CoA carboxylase activity was also affected in an erratic manner by the compounds. Compounds **1,5, 6** and **7** resulted in 44% to 75% suppression after 60 min at 100 μM. Compounds **4, 9** and **10** had marginal effects and compounds **3** and **8** had no effects on the activity. *sn*-Glycerol-3-phosphate acyl transferase activity was inhibited 48% to 70% by compounds **1, 3, 4, 7, 8** and **10** and 25% to 36% by compounds **5** and **6** but compound **9** had no effect. Phosphatidylate phosphohydrolase activity was markedly reduced greater than 70% at 100 μM with the exception of compound **9** which only caused 57% reduction. Hepatic lipoprotein lipase activity was essentially unaffected by compounds **3, 5, 7, 8** and **9,** and was moderately reduced by compounds **4 ,6** and **10.** Compounds **1** and **2** caused reduction of hepatic lipoprotein lipase activity of 45% to 59% after 60 min incubation

### Discussion

The observed reduction in the activities of the hepatic regulatory enzymes caused by the thiosemicarbazones and their metal complexes are of a magnitude to account for the reductions of serum cholesterol and triglyceride levels after 16 days *in vivo.* The reduction of acetyl CoA synthetase
and ATP dependent citrate lyase activities not only would reduce the synthesis of cholesterol in the cytoplasm of the cell but would also lower the levels of fatty acids and neutral lipids since acetyl-CoA is the common starting intermediate for all of the synthetic pathoways for these lipids. The fact that there was a positive correlation between the ability of the compounds to reduce the activity of HMG-CoA reductase and its ability to lower serum cholesterol levels indicates that this may be a key target of the thiosemicarbazones and their metal complexes.
Reduction of cholesterol-7α–hydroxylase activity by a few compounds would lead to less cholesterol being converted to bile acids for biliary excretion. For the few compounds that inhibited acyl-CoA cholesterol acyl transferase activity less cholesterol esters would be stored in the liver. If the analogous reaction occurred in aorta foam cells then reducing cholesterol esters would reduce the size of the plaque.
Table 2: The Effects of Thiosemicarbazones on Sprague Dawley Liver Enzyme Activities

| Assay | Control | Compound 1 | | | Compound 2 | | |
|---|---|---|---|---|---|---|---|
| (N= 6) | | 25μM | 50μM | 100μM | 25μM | 50μM | 100μM |
| Acetyl CoA Synthetase[a] | 100±5 | 69±5* | 47±4* | 20±2* | 99±6 | 63±3* | 47±4* |
| ATP dependent Citrate Lyase[b] | 100±4 | 65±4* | 51±4* | 38±3* | 48±4 | 36±4* | 30±3* |
| HMG-CoA reductase[c] | 100±6 | 87±5 | 87±6 | 80±4* | 80±4* | 61±4* | 57±5* |
| Cholesterol-7α hydroxylase[d] | 100±7 | 104±6 | 89±5 | 79±5* | 96±6 | 90±5 | 75±5* |
| Acyl-CoA Cholesterol Acyl Transferase[e] | 100±5 | 107±6 | 84±5 | 65±5 | 116±6 | 82±6 | 80±4* |
| Acetyl CoA carboxylase[f] | 100±4 | 61±5* | 51±4* | 37±4* | 68±4* | 63±4* | 56±3* |
| *sn*-Glycerol-3-Phosphate[g] Acyl Transferase[h] | 100±5 | 86±5 | 64±5* | 40±3* | 61±3* | 57±5* | 52±4* |
| Phosphatidylate Phosphohydrolase[i] | 100±6 | 79±4* | 37±4* | 17±3* | 85±6 | 39±3* | 19±2* |
| Hepatic Lipoprotein Lipase[j] | 100±5 | 67±3* | 61±4* | 55±4* | 51±4* | 45±4* | 41±3* |

| Assay | Control | Compound 7 | | | Compound 9 | | |
|---|---|---|---|---|---|---|---|
| (N= 6) | | 25μM | 50μM | 100μM | 25μM | 50μM | 100μM |
| Acetyl CoA Synthetase | 100±5 | 128±8 | 52±5* | 38±4* | 110±7 | 50±5* | 28±4* |
| ATP dependent Citrate Lyase | 100±4 | 52±5* | 39±5* | 35±4* | 97±6 | 94±5 | 67±4* |
| HMG-CoA reductase | 100±6 | 57±4* | 53±5* | 48±3* | 48±4* | 43±3* | 43±3* |
| Cholesterol-7α hydroxylase | 100±7 | 74±3* | 52±4* | 38±4* | 132±6* | 115±5 | 97±6 |
| Acyl-CoA Cholesterol Acyl Transferase | 100±5 | 99±7 | 82±5 | 67±4* | 146±8* | 123±6* | 97±5 |
| Acetyl CoA carboxylase | 100±4 | 54±5* | 44±4* | 40±2* | 87±5* | 72±4* | 63±3* |
| *sn*-Glycerol-3-Phosphate Acyl Transferase | 100±5 | 108±6 | 80±5* | 30±3* | 131±5* | 100±6 | 97±5 |
| Phosphatidylate Phosphohydrolase | 100±6 | 58±5* | 28±3* | 19±2* | 129±6* | 60±5* | 43±4* |
| Hepatic Lipoprotein Lipase | 100±5 | 112±5 | 84±5 | 83±4 | 98±6 | 95±5 | 89±6 |

a = 10.1 mg acetyl CoA formed/ g wet tissue; b = 9.2 mg citrate hydrolyzed/g wet tissue; c = 103,020 dpm/g wet tissue; d = 289, 450 dpm/g wet tissue; e= 86,640 dpm/g wet tissue; f = 43021 dpm/g wet tissue; g = 87, 620 dpm/g wet tissue; h = 11 μg Pi released/g wet tissue;i = 234,675 dpm/g wet tissue.

Table 3 The Effects of Nickel Complexes of Thiosemicarbazones On Sprague Dawley Liver Enzyme Activities

| Assay | Control | Compound 3 | | | Compound 4 | | |
|---|---|---|---|---|---|---|---|
| (N= 6) | | 25μM | 50μM | 100μM | 25μM | 50μM | 100μM |
| Acetyl CoA Synthetase[a] | 100±5 | 46±4* | 31±4* | 19±3* | 98±5 | 48±4* | 10±2* |
| ATP dependent Citrate Lyase[b] | 100±4 | 69±5* | 49±3* | 39±2* | 80±4* | 58±3* | 47±3* |
| HMG-CoA reductase[c] | 100±6 | 65±4* | 53±3* | 42±4* | 76±4* | 48±3* | 48±4* |
| Cholesterol-7α hydroxylase[d] | 100±7 | 93±5 | 76±3* | 62±3* | 112±6 | 97±5 | 84±4 |
| Acyl-CoA Cholesterol Acyl Transferase[e] | 100±5 | 116±6 | 88±5 | 77±5 | 88±5 | 87±5 | 82±4 |
| Acetyl CoA carboxylase[f] | 100±4 | 115±6 | 96±5 | 87±6 | 102±5 | 92±6 | 72±4* |
| *sn*-Glycerol-3-Phosphate[g] Acyl Transferase[h] | 100±5 | 56±4* | 54±3* | 51±3* | 67±3* | 60±6 | 46±3* |
| Phosphatidylate Phosphohydrolase[i] | 100±6 | 70±3* | 29±3* | 23±3 | 100±4 | 46±4* | 21±3* |
| Hepatic Lipoprotein Lipase[j] | 100±5 | 129±7 | 90±6 | 90±6 | 104±5 | 97±6 | 73±5* |

| Assay | Control | Compound 5 | | | Compound 6 | | |
|---|---|---|---|---|---|---|---|
| (N= 6) | | 25μM | 50μM | 100μM | 25μM | 50μM | 100μM |
| Acetyl CoA Synthetase | 100±5 | 121±5 | 67±4* | 42±4* | 72±4* | 48±4* | 32±3* |
| ATP dependent Citrate Lyase | 100±4 | 93±6 | 65±5* | 53±5* | 80±6 | 54±3* | 41±2* |
| HMG-CoA reductase | 100±6 | 67±5* | 57±5* | 56±4* | 66±4* | 42±4* | 40±3* |
| Cholesterol-7α hydroxylase | 100±7 | 107±6 | 88±6 | 76±5* | 89±6 | 81±4 | 59±4* |
| Acyl-CoA Cholesterol Acyl Transferase | 100±5 | 89±7 | 87±6 | 83±5 | 122±5 | 99±6 | 86±5 |
| Acetyl CoA carboxylase | 100±4 | 45±4* | 35±3* | 25±4 | 49±4* | 42±4* | 42±3* |
| *sn*-Glycerol-3-Phosphate Acyl Transferase | 100±5 | 124±5 | 107±6 | 75±4* | 116±6 | 66±5* | 64±3* |
| Phosphatidylate Phosphohydrolase | 100±6 | 44±3* | 25±4* | 19±3* | 48±3* | 26±3* | 13±2* |
| Hepatic Lipoprotein Lipase | 100±5 | 149±7* | 140±8* | 95±6 | 89±4 | 83±5 | 73±4* |

The reduction of acetyl CoA carboxylase activity by the thiosemicarbazones and their metal complexes would lead to less synthesis of fatty acids and the suppression of *sn*-glycerol-3-phosphate acyl transferase activity would lead to less neutral lipid synthesis. The ability of the compounds to lower phosphatidylate phosphohydrolase activity would block the removal of the phosphorus moiety from phospholipids so that triglyceride could be formed. This again appears to be major target of the agents in lowering serum triglyceride levels.

Interestingly there did not appear to be any differences in the effects of thiosemicarbazones compared to their metal complexes on either the serum lipid levels or the enzyme activities. Nor did any one type of metal complex appear to be better in reducing these parameters compared to another. The amine carboxyboranes and their metal complexes also demonstrated a pattern of having multiple target enzymes within *de novo* lipid synthesis [1-3]. These derivatives had more effect on acyl-CoA cholesterol acyl transferase activity than the thiosemicarbazones and its metal complexes while the thiosemicarbazones and their metal complexes had more effects on HMG-CoA reductase activity than the amine carboxyboranes and their metal complexes. Similar effects were observed on the other enzyme activities examined by both groups of compounds as well as the magnitude of serum cholesterol and triglyceride reduction in rodents.

Table 4 The Effects of Cobalt Complexes of Thiosemicarbazones On Sprague Dawley Liver Enzyme Activities

| Assay | Control | Compound **8** | | | Compound **10** | | |
|---|---|---|---|---|---|---|---|
| (N= 6) | | 25μM | 50μM | 100μM | 25μM | 50μM | 100μM |
| Acetyl CoA Synthetase[a] | 100±5 | 83±5* | 57±4* | 36±3* | 53±4* | 33±3* | 20±3* |
| ATP dependent Citrate Lyase[b] | 100±4 | 63±4* | 39±3 | 35±3* | 111±5 | 68±4* | 43±3* |
| HMG-CoA reductase[c] | 100±6 | 54±5* | 48±3* | 32±4* | 56±5* | 46±4* | 45±4* |
| Cholesterol-7α hydroxylase[d] | 100±7 | 79±6* | 71±4* | 57±4* | 118±6 | 108±5 | 93±5 |
| Acyl-CoA Cholesterol Acyl Transferase[e] | 100±5 | 101±5 | 74±5* | 61±4* | 110±5 | 84±5 | 77±3* |
| Acetyl CoA carboxylase[f] | 100±4 | 70±4* | 96±5 | 87±6 | 102±5 | 92±6 | 72±4* |
| *sn*-Glycerol-3-Phosphate[g] Acyl Transferase[h] | 100±5 | 56±4* | 54±3* | 51±3* | 67±3* | 60±4* | 46±3* |
| Phosphatidylate Phosphohydrolase[i] | 100±6 | 70±5* | 29±3* | 23±3* | 100±4 | 46±4* | 21±3* |
| Hepatic Lipoprotein Lipase[j] | 100±5 | 129±7* | 90±5 | 90±6 | 104±5 | 97±6 | 73±5* |

### References

1. I.H. Hall, B.F. Spielvogel, A. Sood, K.W. Morse, V. M. Noewood, III, O.T. Wong, *Metal Based Drugs***1**, 329-336, 1994.
2. I.H. Hall, W.L. Williams, Jr.,C.J. Gilbert, A.T. McPhail, and B.F. Spielvogel, *J. Pharm. Sci.* 73, 973-977, 1984.
3. I H. Hall, B.S. Burnham, S.Y. Chen, A. Sood, B.F. Spielvogel, and K.W. Morse, *Metal Based Drugs* 2, 1-12, 1995.
4. I. H. Hall, B.F. Spielvogel, T.S. Griffin, E.L. Docks, R.J. Brotherton, *Res. Commun. Chem. Path. & Pharmacol* **65**, 297-314, 1989.
5 I.H. Hall, B.F. Spielvogel, A. Sood, F. Ahmed, S. Jafri, *J. Pharm. Sci.* **76**, 359-365, 1987.
6. A. Sood, C.K. Sood, B.F. Spielvogel, I.H. Hall, I.H., *Eur. J. Med. Chem.* **25**, 301-308, 1990.
7. D.X. West, A.E. Liberta, K.G. Rajendran, and I.H. Hall, *Anti-Cancer Drugs* **4**, 241-249, 1993.
8. I.H. Hall, K.G. Raijendra, D.X. West, A.E. Liberta, *Anti-Cancer Drugs* **4**, 231-240, 1993.
9. I.H. Hall, S.Y. Chen, K.G. Rajendran, and D.X. West, *Appl. Organomet Chem.***10**, 485-493,1996.
10. A.T. Ness, J.V. Pastewka, A.C. Paecock, *Clin. Chim. Acta* **10**, 229-237, 1964.
11. I.H Hall, O.T. Wong, R. Simlot, D.J. Reynolds, *Pharm. Res.* 9, 1324-1329, 1992.
12. M. Hoffman, L. Weiss, O.H. Wieland, *Anal. Biochem.* **84**, 441-448, 1978.
13. A.G. Goodridge, *J. Biol. Chem.* **248**, 4318-4327, 1973.
14. G.T. Haven, J.R. Kremian, T.T. Nguyen, Res. Commun, *Chem. Pathol. Pharmacol.* **6**, 253-261, 1973.
15. F. Wada, K. Hirata, Y. Sakameto, *J. Biochem [Tokyo],* **65**, 171-175, 1989.
16. S. Balsasbramaniam, K.A. Mitropoulos, S. Venkatean, *Eur. J. Biochem.* **90**, 377-383, 1978.
17. S. Shefer, S. Hauser, E.H. Mosbach, *J. Lipid Res.* **19**, 467-477, 1978.
18. M.D. Greenspan, J.M. Lowenstein, *J. Biol. Chem.* **243**, 6273-6280, 1968.
19. R.G. Lamb, S.D. Wyrick, C. Piantadosi, *Artherscol.* **27**, 147-154, 1977.
20. R.D. Mavis, N. Jacob, J.N. Finkelstein, B.P. Hall, *J. Lipid Res.* **19**, 467-477, 1978.
21. A. Chait P.h. Iverius, J.D. Brunzell, *J. Clin. Invest.* **69**, 490-493, 1982.

**Received: March 22, 1999 - Accepted: April 23, 1999 - Received in revised camera-ready format: April 24, 1999**

